# Soil bacterial communities associated with multi-nutrient cycling under long-term warming in the alpine meadow

**DOI:** 10.3389/fmicb.2023.1136187

**Published:** 2023-02-23

**Authors:** Xiaorong Zhou, Xianke Chen, Xiangning Qi, Yiyuan Zeng, Xiaowei Guo, Guoqiang Zhuang, Anzhou Ma

**Affiliations:** ^1^Research Center for Eco-Environmental Sciences, Chinese Academy of Sciences, Beijing, China; ^2^College of Resources and Environment, University of Chinese Academy of Sciences, Beijing, China; ^3^Sino-Danish College of University of Chinese Academy of Sciences, Beijing, China; ^4^Sino-Danish Center for Education and Research, Beijing, China; ^5^Key Laboratory of Adaptation and Evolution of Plateau Biota, Northwest Institute of Plateau Biology, Chinese Academy of Sciences, Xining, China

**Keywords:** climate warming, profile, multi-nutrient cycling, keystones, bacteria

## Abstract

**Introduction:**

The functions of terrestrial ecosystems are mainly maintained by bacteria, as a key component of microorganisms, which actively participate in the nutrient cycling of ecosystems. Currently, there are few studies have been carried out on the bacteria contributing to the soil multi-nutrient cycling in responding to climate warming, which hampers our obtainment of a comprehensive understanding of the ecological function of ecosystems as a whole.

**Methods:**

In this study, the main bacteria taxa contributing to the soil multi-nutrient cycling under the long-term warming in an alpine meadow was determined based onphysichemical properties measurement and high-throughput sequencing, and the potential reasons that warming altered the main bacteria contributing to the soil multi-nutrient cycling were further analyzed.

**Results:**

The results confirmed that the bacterial β-diversity was crucial to the soil multi-nutrient cycling. Furthermore, Gemmatimonadetes, Actinobacteria, and Proteobacteria were the main contributors to the soil multi-nutrient cycling, and played pivotal roles as keystone nodes and biomarkers throughout the entire soil profile. This suggested that warming altered and shifted the main bacteria contributing to the soil multi-nutrient cycling toward keystone taxa.

**Discussion:**

Meanwhile, their relative abundance was higher, which could make them have the advantage of seizing resources in the face of environmental pressures. In summary, the results demonstrated the crucial role of keystone bacteria in the multi-nutrient cycling under the climate warming in the alpine meadow. This has important implications for understanding and exploring the multi-nutrient cycling of alpine ecosystems under the global climate warming.

## 1. Introduction

Ecosystems maintain multiple functions simultaneously. Nutrient cycling is an important part of ecosystem function. The index of multi-nutrient cycling defined by humans, which is not a single measurable index that can quantify more than one process of the ecosystem (Byrnes et al., 2014). Microbes are the engines driving the biogeochemical cycle of the earth, especially in the terrestrial ecosystems (Falkowski et al., 2008), and play a vital role in the function of ecosystems. Numerous studies have focused on the role of microorganisms in the multifunctionality of ecosystems (Wagg et al., 2014; Jiao et al., 2019, 2021). It has been found that the biodiversity of microbes clearly influenced the ecosystem multifunctionality (Jing et al., 2015). The multifunctionality was driven by soil microbial diversity, which has been demonstrated by previous studies in terrestrial ecosystems (Jing et al., 2015; Delgado-Baquerizo et al., 2016). In addition, species themselves also affect the function of ecosystems. The functional importance of a single species does not directly translate into the functional importance of the microorganisms in the community as a whole (Pasari et al., 2013). This is because there are interactions between species. The research demonstrated that the positive and negative associations between species were tightly correlated with the ecosystem function (Jiao et al., 2021). Thus, it is necessary to pay attention to the role of microorganisms in the microbial network, especially the keystone nodes in the network which may have a more dramatic impact on the ecosystem multifunctionality. Soil microbial communities account for the majority of belowground biodiversity, particularly bacterial communities, make significant contributions to ecosystem biomass, biodiversity, element cycling, and energy flow (Nielsen et al., 2015; Jiao et al., 2019). However, little attention has been paid to the impact of bacterial communities on the multi-nutrient cycling under climate change. Due to the importance of multifunctionality, it is necessary to determine the contribution of bacteria to the soil multi-nutrient cycling in terrestrial ecosystems.

In recent years, evidence has emerged that terrestrial ecosystems have a positive feedback on climate warming (Heimann and Reichstein, 2008). Specifically, climate warming effected the activities of soil microorganisms directly and indirectly, and greenhouse gases (e.g., carbon dioxide, methane) produced by microbial activities were released into the atmosphere, which made a contribution to global warming (Petchey et al., 1999; Bardgett et al., 2008; Vincent, 2010; Dove et al., 2021). The soil multi-nutrient cycling mainly mediated by microbes, so the effect of climate warming on microbiomes will ultimately affect soil multi-nutrient cycling. The bacterial diversity has responded differently to climate warming. It was found that bacterial α-diversity in subtropical planted forest topsoil (0–10 cm; Wang et al., 2019) and alpine meadow topsoil (0–20 cm; Li et al., 2016) increased under climate warming. However, there was no significant change in bacterial α-diversity in the topsoil (0–10 cm) of the forest-alpine cross zone after warming (Zheng et al., 2020). Thus, differences in the α-diversity of bacteria responding to warming may affect the contribution of bacteria populations to soil multi-nutrient cycling in the ecosystem, owing to previous studies that have proved that the diversity of bacteria was tightly correlated with the multi-nutrient cycling. Warming also affected the bacterial population changes besides the bacterial diversity (Guo et al., 2018; Dove et al., 2021; Zhou et al., 2021). The composition of soil bacteria have been altered by warming (DeAngelis et al., 2015; Cheng et al., 2017). For instance, there has been a decline in the relative abundance of Planctomycetes and Actinobacteria (Zhou et al., 2021). Climate warming enhanced the interactions among species, owing to the increased complexity and stability of microbial networks (Yuan et al., 2021). Connectors in microbial networks were more connected to other modules (Guimerà and Nunes Amaral, 2005). Therefore, these species were more resistant to environmental stresses. However, few researches focused on the changes in bacterial community composition affecting the roles of bacteria in soil multi-nutrient cycling. Furthermore, bacterial communities are present throughout soil profile. There is little further attention has been paid to the impact of population changes on the multi-nutrient cycling under warming across the entire soil profile. It remains unclear which bacterial groups contribute to the multi-nutrient cycling, particularly under long-term warming.

More than 85% of the Qinghai-Tibetan Plateau is covered by alpine meadows, shrub-meadows, and alpine steppes, and the rate of increase in its temperature is approximately three times that of global warming (Qiu, 2008). Particularly, the alpine meadow ecosystem is extremely sensitive and vulnerable to climate change, which is very important for the dynamic changes in the element cycling (Chen et al., 2013). Thus, the Qinghai-Tibetan Plateau is a typical experimental area for exploring the response and feedback of subsurface bacterial communities to climate warming (Heimann and Reichstein, 2008). It is very necessary to explore the effects of climate warming on bacteria involved in soil multi-nutrient cycling in alpine ecosystems that are extremely sensitive to climate warming. The aim of this study was to identify the potential association between the bacterial community and soil multi-nutrient cycling, and to investigate whether warming affected the main bacteria contributors to the multi-nutrient cycling in the alpine meadow. The importance of bacterial diversity to the soil multi-nutrient cycling was first explored. Then, the main bacteria involved in the multi-nutrient cycling were identified under control and warming. This study deepened our understanding of the bacteria involved in soil multi-nutrient cycling under climate warming, especially in alpine ecosystems.

## 2. Materials and methods

### 2.1. Study site and sampling

This study was conducted at the Haibei National Alpine Grassland Ecosystem Research Station (37°36′38.53″ N, 101°18′49.31″ E), in northwestern China at an average elevation of 3,195 m. During the summer, the station area has a southeast monsoon climate, and in winter, the northwest Asian cold effects it. The warm seasons are short and cool, and the cold seasons are long and cold. The annual maximum and minimum extreme temperatures are 27.6°C and −37.1°C, respectively. The annual average temperature is −1.7°C, and the annual precipitation is 426–860 mm, of which 80% is distributed in the plant growth period from May to September. The parent material of the soil is loess with alluvial sediments underneath. The open top chambers (OTCs) were often used to explore the responses of ecosystems to climate warming (Marion et al., 1997). In this study, these OTCs were 1.5 m in diameter and 40 cm in height. The temperature of the OTCs was measured by online monitoring device. During the growing season, OTCs increased the average daily temperature by 0.6–2.0°C, and more information about OTCs referred to the reference (Klein et al., 2004). In August 2021, the depth of the soil core collected in the study area was 0–80 cm. Nine soil cores (core diameter of 38 mm) were randomly collected at one time in the warming (soil in OTCs) and control (soil outside OTCs) plots using a soil sampling device, respectively (Supplementary Figure S1). Meanwhile, the number of soil samples replicates is nine. The cores were manually segmented as follows: 0–15 cm, 15–30 cm, 30–45 cm, 45–60 cm, and 60–80 cm (corresponding to L1, L2, L3, L4, and L5, respectively). Consequently, there were 90 (2 × 9 × 5) samples in total. After the soil samples were transported to the laboratory, some were stored at 4°C to measure the physicochemical properties, while other parts were stored at −20°C for other experiments.

### 2.2. The assessment of multi-nutrient cycling index

The contents of ammonium nitrogen (NH_4_^+^-N) and nitrate nitrogen (NO_3_^−^-N) were measured using an AA3 continuous flow analytical system (Seal, German). The soil organic carbon (SOC) concentration was determined using the potassium dichromate oxidation method (Walkley and Black, 1934). The soil dissolved organic carbon (DOC) was extracted by adding 10 mL of water to 1 g of soil, shaking for 24 h at 30°C and filtering through a 0.45 μm filter (Millipore). The sample was analyzed using an elementary total organic carbon (TOC) analyzer (Vario TOC; Elementar, Germany). The total carbon (TC) and total nitrogen (TN) content were measured using an elementary analyzer (Vario MACRO Cube, Elementar, Germany), while the total phosphorus (TP) content was measured using the acid fusion-Mo-Sb anti spectrophotometric method. The available nitrogen (AN) concentration was determined using the alkali diffusion method. Finally, the available phosphorus (AP) was extracted using sodium bicarbonate and was measured using the molybdenum-blue method (Huang et al., 2021).

The concept of soil multi-nutrient cycling has been applied in many studies (Pasari et al., 2013; Delgado-Baquerizo et al., 2016; Gross et al., 2017; Le Bagousse-Pinguet et al., 2019; Zheng et al., 2019). First, we standardized each of the nine measured functions (NH_4_^+^-N, NO_3_^−^-N, DOC, TN, TC, TP, SOC, AN, and AP) using the Z-score transformation, and then, we averaged them to obtain an index of the multi-nutrient cycling for each site (Delgado-Baquerizo et al., 2016). We chose these functions because they provided some of the essential functions needed to support and regulate ecosystems.

### 2.3. DNA extraction and sequencing

The total deoxyribonucleic acid (DNA) in the soil was extracted from 0.5 g of thoroughly mixed soil using a MOBIO PowerSoil DNA isolation kit (MOBIO Laboratories, Carlsbad, CA, United States) according to the instructions of the kit manufacturer. The integrity and purity of the DNA were monitored on a 1% agarose gel. The V3–V4 hypervariable regions of 16S ribosomal ribonucleic acid (rRNA) genes were PCR amplified with primers 338F (5′-ACTCCTACGGGAGGCAGCA-3′) and 806R (5′-GGACTACHVGGGTWTCTAAT-3′). Polymerase chain reactions (PCRs) were conducted in a 50 μL reaction system, containing 25 μL 2Χ Premix Taq (TaKaRa), 1 μL each of 10 μM forward and reverse primers, and 50 ng of the DNA. The thermal cycle conditions were as follows: 5 min at 94°C for initialization; followed by 30 cycles of 30 s of denaturation at 94°C, 30 s of annealing at 52°C, and 30 s of extension at 72°C, 10 min final elongation at 72°C, and then held at 4°C. The PCR products were tested using 1% agarose gels and purified using an E.Z.N.A. Gel Extraction Kit (Omega). The concentrations were measured using NanoDrop 2000 and Qubit 3.0 (Thermo Fisher Scientific, Waltham, United States) and were stored at −80°C. The DNA samples were then sequenced using an Illumina Nova6000 platform and 250 bp paired-end reads were generated (Guangdong Magigene Biotechnology Co., Ltd. Guangzhou, China).

### 2.4. Bioinformatics and statistical analyses

A pipeline was used to generate an operational taxonomic unit (OTU) table^1^ (Feng et al., 2017). Wilcoxon test was used to compare whether α-diversity was significantly different between treatments. Principal coordinates analysis (PCoA) was conducted to assess the community similarity among the depth intervals. Significant differences in the composition of the community between depth intervals were assessed using permutation multivariate analysis of variance (PERMANOVA), with 999 permutations calculated per test.

After analysis by the online pipeline,^2^ phylogenetic molecular ecological network (pMEN) graphs were created using the Gephi 0.9.2 software program (Zhou et al., 2010; Deng et al., 2012). The OTUs appearing in more than half of the samples were retained, and the RMT threshold were consistent (0.830). Then the Spearman correlation matrix were used to construct the microbial network. The roles of the network nodes were identified based on their values of within-module connectivity (Zi) values and among-module connectivity (Pi) values (Guimerà and Nunes Amaral, 2005). Furthermore, the Levins’ niche breadth index was estimated according to the reference (Jiao et al., 2021).

In addition, the random forest (RF) algorithm was used to identify the taxa that represented the most under warming and control using the rfPermute package. The A3 package was applied to calculate the significance level and cross-validated R^2^ value of the model with 5,000 permutations of the response variable (Jiao et al., 2020).

All of the statistical analyses were carried out in the R environment (v4.0.4; https://www.r-project.org/), using vegan, ggplot2, and spaa.

## 3. Results

### 3.1. The diversity of bacteria correlated with nutrient variables

**Figure 1:**
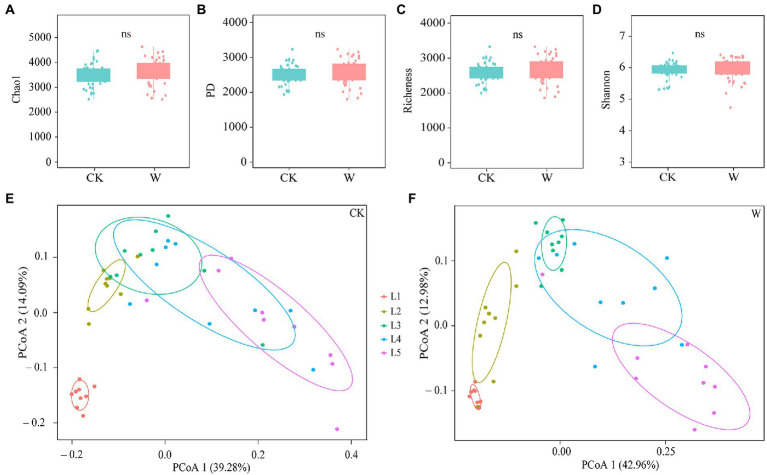
The bacterial diversity throughout the entire soil profile under control and warming. The α-diversity of bacteria between control and warming (using the Mann–Whitney *U*-test) **(A–D)**. PCoA analysis was performed on differences in community composition at different depths under control **(E)** and warming **(F)**.

There was a total of 11,463,321 reads were obtained, and they were grouped into 34,192 OTUs with 97% similarity. The species accumulation curves were close to the asymptotes, suggesting that the sequencing recovered much of the local species diversity (Supplementary Figure S2). First, warming had no significant effect on the α-diversity of the bacterial community (Figures 1A–D). The Shannon index of bacteria in L1 increased after warming, indicating that the diversity of the community increased (Supplementary Figure S3D). Then, the analysis of PCoA was used to explore the overall variability of the bacteria composition (Figures 1E,F), which that about 54% of the variance could be explained by depth, indicating that warming significantly increased the differences among the bacterial communities in the soil profile. Meanwhile, warming significantly increased the differences between bacterial communities in L1, L2, and L3 layers (Supplementary Figures S4A–C).

**Figure 2:**
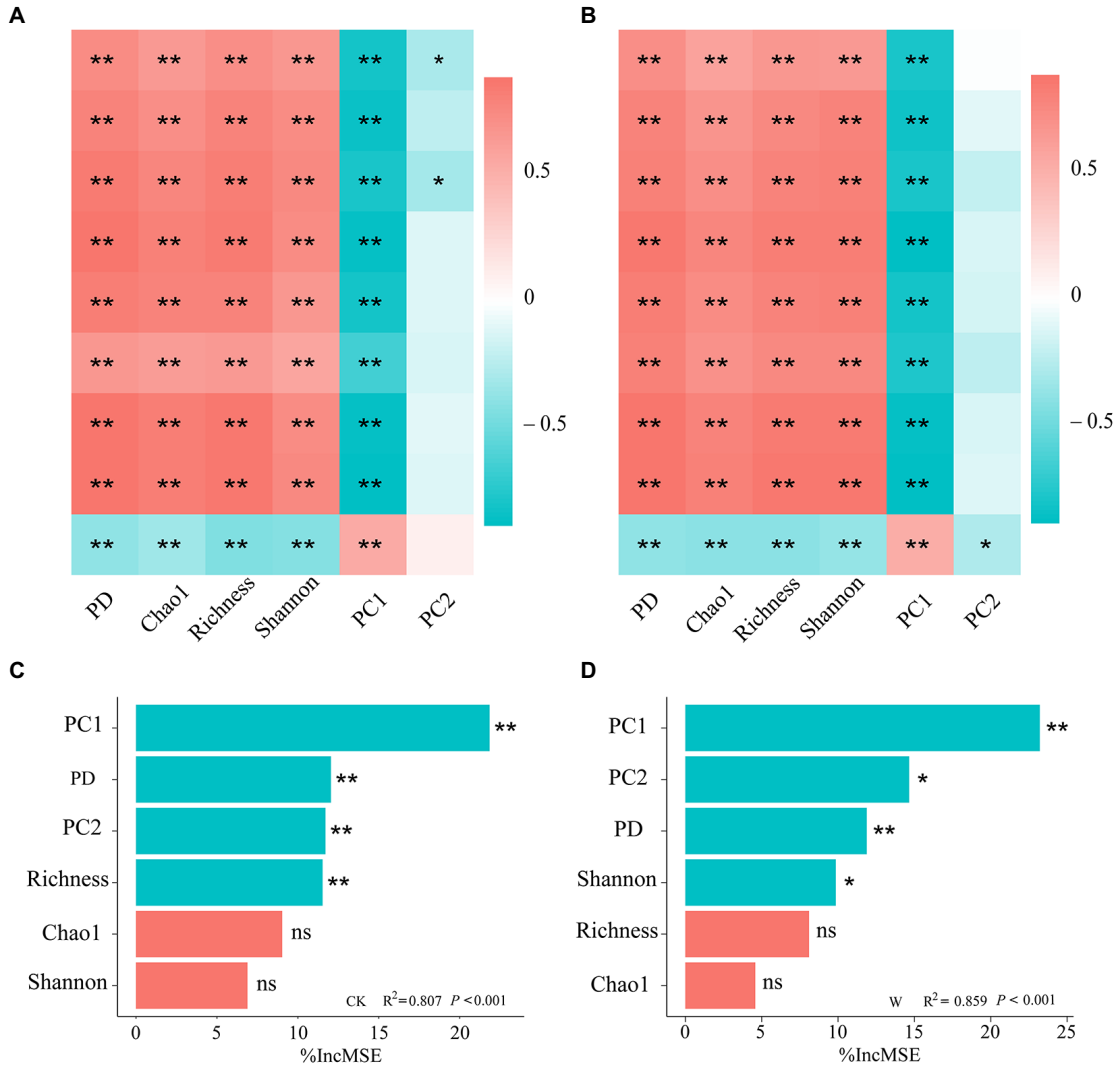
The correlation between nutrient variables and the diversity of bacteria in the alpine ecosystem. Heatmaps of the Spearman’s correlation coefficients between community diversity and individual nutrient variables under control **(A)** and warming **(B)**. ^*^p < 0.05; ^**^p < 0.01. The importance of bacterial α-and β-diversity driving the cycling of soil multi-nutrient in the entire profile under control **(C)** and warming **(D)**, was assessed by RF analysis.

To explore the relationship between bacterial diversity and the individual nutrient variables, the results of their correlation coefficients were shown by a correlation heatmap (Figures 2A,B). Both α-diversity (PD, Chao 1, Richness, Shannon) and β-diversity (PC1) of bacteria were significantly correlated with all individual variables of nutrient in the whole soil profile (Figures 2A,B), indicating that both diversity and community composition of bacteria play crucial roles in soil multi-nutrient cycling. Most indexes of α-diversity were significantly positively correlated with individual nutrient variables except AP, while the β-diversity was significantly negatively correlated. Warming appeared to have no effect on the correlation of bacterial diversity with individual nutrient variables.

Bacterial diversity was significantly correlated with multiple soil nutrient variables (Figures 2A,B), but the ecosystems maintain multiple functions at the same time, the index of multi-nutrient cycling could better reflect the functions of terrestrial ecosystems. Therefore, it is of great significance to explore the importance of bacterial diversity to the cycling of soil multi-nutrient. Based on the random forest analysis, the main bacterial predictors of the soil multi-nutrient cycling index were identified, which elucidated the major factors driving the soil multi-nutrient cycling. The results found that most essential variable for predicting multi-nutrient cycling across the entire soil profile was the β-diversity, followed by the α-diversity (Figures 2C,D). It was worth noting that the bacterial diversity index related to the change in soil multi-nutrient cycling index differed after warming, although this difference was little obvious.

### 3.2. Potential bacteria contribute to the soil multi-nutrient cycling in the alpine meadow

**Figure 3:**
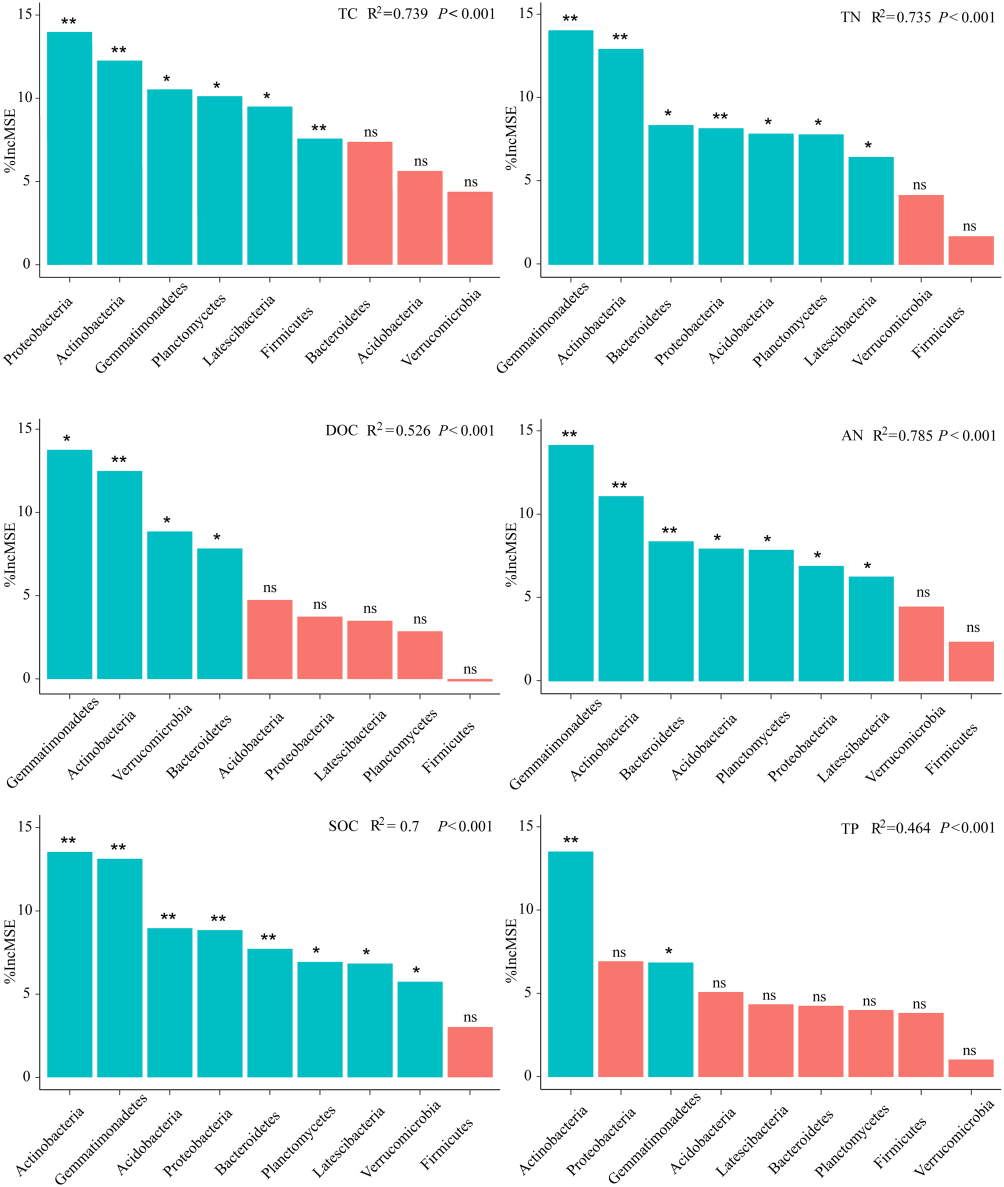
Main potential bacteria contribute to soil multi-nutrient cycling in the alpine ecosystem under warming. The importance of these predictors was estimated using the percent increase in the MSE of the variables, with higher MSE% values indicating more important predictors. The *p* < 0.05 (*) and *p* < 0.01 (**) indicate significance at the levels of 5% and 1%, respectively.

The biological contribution of the bacteria to the soil properties was further assessed at the phylum level (dominant phyla) based on the RF analysis (Figure 3; Supplementary Figure S5). It is evident that rather than all bacterial phyla made the same contribution to various soil variables. Specifically, the most important variable to predict the soil properties was Planctomycetes (TC, SOC, TN, and AN), indicating that they were crucial to soil nutrient cycling (*p* < 0.001). In addition, other important variables for predicting the soil properties were the Gemmatimonadetes for TP (*p* = 0.001), and the Verrucomicrobia for DOC (*p* < 0.001; Figure 3). In addition, after warming, Gemmatimonadetes was the most important to predict the soil properties (DOC, SOC, TN, and AN), followed by Actinobacteria. Proteobacteria was a more important variable for predicting TC and AP under warming (*p* < 0.001). Planctomycetes and Verrucomicrobia played potential roles in multi-nutrient cycling in the alpine meadow (Supplementary Figure S5). However, these roles were filled by Gemmatimonadetes, Actinobacteria, and Proteobacteria after warming (Figure 3), suggesting that warming shaped the main bacteria contributing to the soil multi-nutrient cycling throughout the soil entire profile.

### 3.3. Species coexistence of bacteria and their biomarkers in the entire soil profile

**Figure 4:**
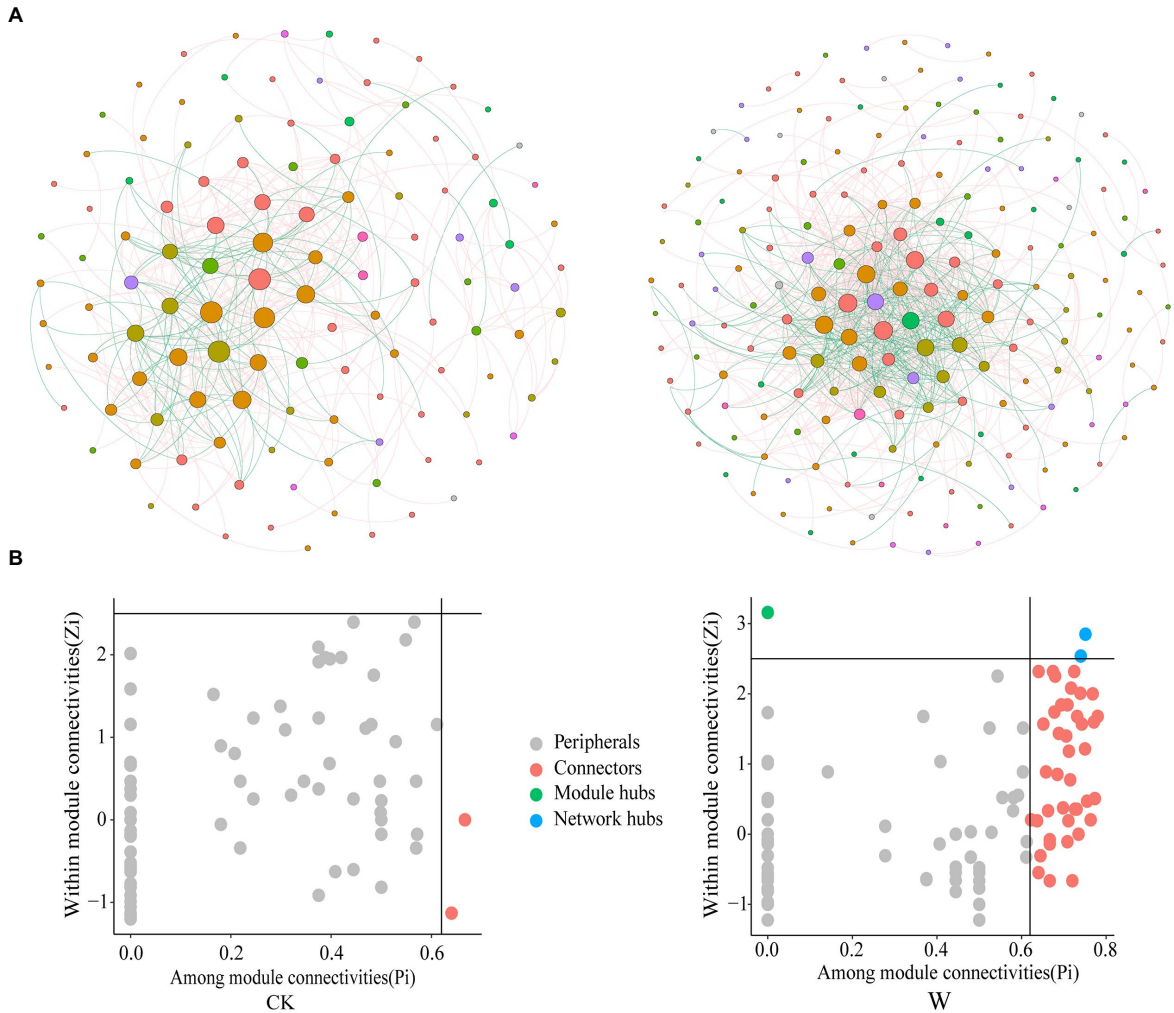
The bacteria network and biomarkers under control and warming. **(A)** Microbial co-occurrence networks under control and warming. The thickness of the edges is proportional to the value of the Spearman’s correlation coefficient, and the size of the nodes is proportional to the degree of the OTUs. **(B)** Roles of nodes based on their Zi and Pi values in the co-occurrence networks.

Phylogenetic molecular ecological network (pMEN) often used to study the interaction between species (Deng et al., 2012). The coexistence of species in soil was investigated by constructing microbial co-occurrence networks (Figure 4A), owing to the existence of bacteria throughout the entire profile. The number of nodes for control and warming were 120 and 192, respectively; and the number of edges were 431 and 831. In addition, the total nodes, links, and average degree increased after warming (Supplementary Table S1), indicating that the bacteria network became more complex and connected. The proportion of negative interactions also increased (Supplementary Table S1). Due to environmental stress caused by warming, competitive interactions between species has increased, which may lead to a decline in community diversity. Furthermore, the roles of bacterial taxa were also determined based on their Zi and Pi values in the bacteria networks. The proportion and number of keystone nodes (i.e., connectors, network, and module hubs) increased after warming, and were associated with Acidobacteria, Actinobacteria, Bacteroidetes, Proteobacteria (Figure 4B; Supplementary Table S2).

**Figure 5:**
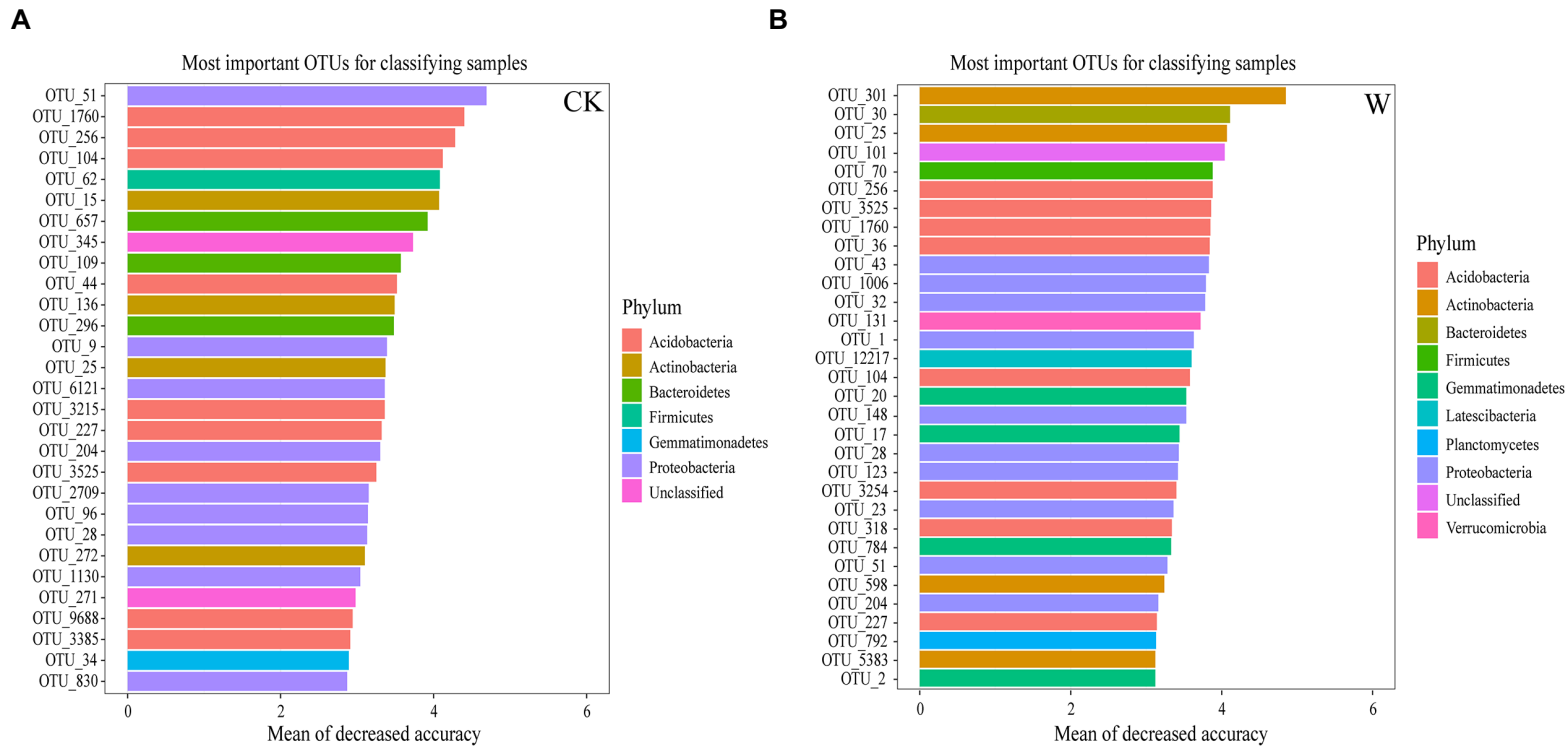
Biomarkers of bacteria between control and warming throughout the entire profile. The top 29 biomarker bacterial OTUs were accurately identified by RF analysis in the control **(A)**, and 32 were identified in the warming profile **(B)**.

Generally, there are some microorganisms that are particularly sensitive to changes in the environment, which could be regarded as biomarkers. The biomarkers were identified throughout the whole soil profile based on the analysis of RF. There were 29 OTUs were biomarkers, and most of them belonged to Acidobacteria, Actinobacteria, Bacteroidetes, and Proteobacteria (Figure 5). In addition, Verrucomicrobia, Planctomycetes, Firmicutes, Gemmatimonas, Roseisolibacter, Dissulfurirhabdus, Solimonas, Sulfuricaulis, and Thiobacter could be considered to be biomarkers of the entire soil profile after warming (Supplementary Table S3). Moreover, some specific biomarkers (OTUs) were also found to play important roles in the network under warming. Some bacteria in Acidobacteria (OTU_104, OTU_1760, OTU_256, and OTU_36), Gemmatimonadetes (OTU_17 and OTU_20), Actinobacteria (OTU_598), Proteobacteria (OTU_1, OTU_43 and OTU_51), and Verrucomicrobia (OTU_131) were the key connectors and module hubs in the bacterial network under warming (Supplementary Tables S2, S3). Therefore, these OTUs with dual identities could be regarded as species that are more sensitive and even more strongly affected by climate warming.

## 4. Discussion

Microorganisms actively participate in the cycling of various nutrient elements, which is of great significance to the soil multi-nutrient cycling in terrestrial ecosystems. The diversity of microorganisms is closely related to the nutrient variables, whether they are single variables or multi-nutrient cycling index. Multiple elemental cycles occur simultaneously in the soil ecosystems. Therefore, the index of multi-nutrient cycling was used to explore the functions of terrestrial ecosystems. Soil microbial communities account for the majority of belowground biodiversity, particularly bacterial communities. Therefore, the roles of bacteria in the soil multi-nutrient cycling were first determined by correlating the bacterial diversity with the multi-nutrient cycling. The results indicated that the bacterial β-diversity had a stronger effect on the soil multi-nutrient cycling throughout the entire soil profile. Most importantly, Gemmatimonadetes, Actinobacteria, and Proteobacteria, which were identified as biomarkers and keystone nodes, were important contributors to the cycling of multi-nutrient in the soil profile after warming. Long-term warming has resulted in keystone taxa being the main bacteria involved in the soil multi-nutrient cycling. This study shed light on the contribution of bacteria to soil multi-nutrient cycling, especially provided insights into the impact of changes in bacterial populations on the soil multi-nutrient cycling in the context of a warming climate.

### 4.1. Main bacteria contributing to the soil multi-nutrient cycling were altered in the whole soil profile after warming

Bacteria are important components in driving biogeochemical cycles in terrestrial ecosystems (Wagg et al., 2014), such as the degradation of organic matter (Xue et al., 2016), and nitrogen cycling (Zhou et al., 2015; Li et al., 2019). The microbial diversity is tightly correlated with the soil multi-nutrient cycling in terrestrial ecosystems (Delgado-Baquerizo et al., 2016; Zheng et al., 2019). In this study, it was found that the bacterial β-diversity was the most important in predicting the soil multi-nutrient cycling index across the entire soil profile (Figures 2C,D). This was supported by the results of a previous study, which reported that the β-diversity was more important when multiple functions were considered (Pasari et al., 2013). Moreover, %IncMSE (percent increase of the mean square error) in PC1 increased from 21.84% to 23.20% after warming (Figures 2C,D), which was consistent with the fact that differences between the bacterial communities along the soil profile increased after warming (Figures 1E,F). The bacterial diversity associated with changes in the soil multi-nutrient cycling index differed after warming (Figures 2C,D), which indicated the importance of bacterial diversity on soil multi-nutrient cycling differed after warming.

Warming altered the bacteria involved in the multi-nutrient cycling, although there was seemed to have less effect on the proportion of bacteria in the whole profile (Supplementary Figure S6A). Planctomycetes and Verrucomicrobia were important contributors to the soil multi-nutrient cycling, but Gemmatimonadetes, Actinobacteria and Proteobacteria became the important contributors after warming (Figure 3; Supplementary Figure S5). Studies have demonstrated that the warming accelerated lignin decomposition caused by Proteobacteria (Tao et al., 2020), which supported the results in this study that Proteobacteria played a crucial role in the multi-nutrient cycling under warming. Actinobacteria exhibited an increasing trend under climate warming (Rui et al., 2015), which meant Actinobacteria could be better able to adapt to warming. In addition, Gemmatimonadetes, as dominant phylum, have a stronger tolerance to environmental stress.

### 4.2. Species coexistence of bacteria and their biomarkers in the whole soil profile

The activities and interspecific interactions of microbiomes affected the soil nutrient cycling and many ecosystem processes (Cardinale et al., 2012), so the role of bacteria was explored in multi-nutrient cycling based on the coexistence of species. Warming increased the complexity of bacterial networks, including the network size, and average degree (Supplementary Table S2), which was consistent with a study in a tallgrass prairie ecosystem (Yuan et al., 2021). Previous studies have found that climate warming has enhanced network stability over time (Yuan et al., 2021). The higher complexity (larger network, higher connectivity) can render the system more resistant (Okuyama and Holland, 2008; Landi et al., 2018) but less resilient (Boucot, 1985). Therefore, it could be speculated that the microorganisms have been adapted to changes in the environment after long-term warming. However, Hernandez et al. found that the increasing stress through anthropogenic perturbations could destabilize microbiomes (Hernandez et al., 2021), suggesting that climate warming has less impact on microbial networks than anthropogenic disturbances. Warming could select some fast-growing bacteria as a deterministic filter (Guo et al., 2018) based on the metabolic theory of ecology (MTE; Brown et al., 2004). The bacteria developed thermal adaptation (Bradford, 2013) to this environmental stress during the long-term warming in this study. This further confirmed the previous conclusion that the microbial community could adapt to the changes in the environment (Walther et al., 2002). The proportion of negative interactions increased after warming, which may lead to a decrease in community diversity or even reduced the multi-nutrient cycling of soil (Delgado-Baquerizo et al., 2016). Most of the keystone nodes were abundant taxa [OTUs with relative abundance above 0.1% of the total sequences (Liu et al., 2015)], and the members of the Betaproteobacteria, Thermoleophilia and Acidobacteria Gp4 classes were the most prominent keystone nodes under warming (Supplementary Table S3), accounting for 52.3% of all of the module hub connectors and network hubs. Some of the biomarkers were keystone nodes in the network under warming (Supplementary Tables S2, S3). It may be the hub species in the network that initially respond to the changes in the environment, sensing and then passing on this information to other species in the population.

Gemmatimonadetes, Actinobacteria, and Proteobacteria were important contributors to the cycling of soil multi-nutrient after warming. Based on this, their roles were further explored in microbial networks and biomarkers. The proportion of Gemmatimonadetes, Actinobacteria, and Proteobacteria in the keystone nodes reached 63.63% under warming (Supplementary Table S2), and their proportion also reached 56.25% in biomarkers after warming (Supplementary Table S3). These results suggested that the main contributors (Gemmatimonadetes, Actinobacteria, and Proteobacteria) serve dual role under warming. They may have been the first to perceive and even adapt to the changes in the environment. In addition, they transmitted this change to their partners through interactions as keystone nodes in the network. Finally, they affected the multi-nutrient cycling of the terrestrial ecosystems.

### 4.3. Bacterial phyla with high relative abundance after warming were the main contributors to multi-nutrient cycling

The potential reasons for the change in bacteria participating in the multi-nutrient cycling after warming were further analyzed from the perspective of relative abundance (Supplementary Figures S6A,B). Planctomycetes and Verrucomicrobia were important contributors to the soil multi-nutrient cycling, and their relative abundance were 0.56% and 2.14%, respectively (Supplementary Figure S6A). The main bacteria that contributed to the soil multi-nutrient cycling became Gemmatimonadetes, Actinobacteria, and Proteobacteria after warming, with the relative abundance 12.83%, 7.06%, and 32.88%, respectively (Supplementary Figure S6A). This indicated that the relative abundance of the three phyla was higher. In order to eliminate the difference in soil heterogeneity caused by depth, the relative abundance of bacteria in each layer was studied (Supplementary Figure S6B). The relative abundance of Gemmatimonadetes, Actinobacteria, and Proteobacteria was higher in each layer. Therefore, the phyla with lower relative abundance are more stressed by warming, which inhibited their functions in soil multi-nutrient cycling, while the phyla with higher relative abundance were more adaptable and better copied with the environmental stress caused by warming. In addition, the community-level niche breadths (*Bcom*) of bacteria in the whole profile showed an increasing trend after warming (Supplementary Figure S6C). It could be explained that the species with high relative abundance have more advantages to occupy resources than the species with low relative abundance, with the increase of niche breadth under warming.

In conclusion, this study filled a gap in the impact of climate warming on bacteria involved in the multi-nutrient cycling. In this study, the diversity and contribution of bacteria were explored to the soil multi-nutrient cycling in an alpine meadow under long-term warming. The results indicated that the bacterial β-diversity played a dominant role in the multi-nutrient cycling in the alpine meadow. In addition, climate warming altered and resulted in keystone taxa being the major bacteria contributing to the soil multi-nutrient cycling. In summary, this study deepens our understanding of bacteria involved in soil multi-nutrient cycling in alpine meadow ecosystems, and shows that keystone bacteria are more essential for the soil multi-nutrient cycling under climate warming. Future studies could further focus on whether metabolic exchange between keystone bacteria affects their roles in soil multi-nutrient cycling under environmental stress of warming.

## Data availability statement

The datasets presented in this study can be found in online repositories. The names of the repository/repositories and accession number(s) can be found in the article/supplementary material.

## Author contributions

XZ, GZ, and AM: conceptualization and design. XZ, XC, and XQ: methodology, data collection and formal analysis. XZ, YZ, and XG: validation, investigation, and resources. XZ: writing the original manuscript. GZ and AM: supervision, reviewing and editing the manuscript. All authors contributed to the article and approved the submitted version.

## Funding

This work was supported by the Second Tibetan Plateau Scientific Expedition and Research Program (2019QZKK0402 and 2019QZKK0307), the National Key Research and Development Program of China (2018YFA0901200), and the National Natural Science Foundation of China (41671270 and 41673082).

## Conflict of interest

The authors declare that the research was conducted in the absence of any commercial or financial relationships that could be construed as a potential conflict of interest.

## Publisher’s note

All claims expressed in this article are solely those of the authors and do not necessarily represent those of their affiliated organizations, or those of the publisher, the editors and the reviewers. Any product that may be evaluated in this article, or claim that may be made by its manufacturer, is not guaranteed or endorsed by the publisher.

## Footnotes

^1^ http://mem.rcees.ac.cn:8080/

^2^ http://ieg4.rccc.ou.edu/MENA/login.cgi
