# Supplementary material for: Soil bacterial communities associated with multi-nutrient cycling under long-term warming in the alpine meadow
Source: Front Microbiol. 2023 Feb 23;14:1136187. doi: 10.3389/fmicb.2023.1136187 (PMC9995882; doi:10.3389/fmicb.2023.1136187)
Supplement: Supplementary file 2 [file Image_1.pdf]

Supplementary Material

Soil bacterial communities associated with multi-nutrient cycling under long-term warming in the alpine meadow

Xiaorong Zhou[1,2], Xianke Chen[1,3,4], Xiangning Qi[1,2], Yiyuan Zeng[1,2], Xiaowei Guo[5], Guoqiang Zhuang[1,2], Anzhou Ma[1,2*]

[1]Research Center for Eco-Environmental Sciences, Chinese Academy of Sciences, Beijing, 100085, China

[2]College of Resources and Environment, University of Chinese Academy of Sciences, Beijing, 100049, China

[3]Sino-Danish College of University of Chinese Academy of Sciences, Beijing, 101400, China

[4]Sino-Danish Center for Education and Research, Beijing, 101400, China

[5]Key Laboratory of Adaptation and Evolution of Plateau Biota, Northwest Institute of Plateau Biology, Chinese Academy of Sciences, Xining, 810008, China

*** Correspondence:** Anzhou Ma: azma@rcees.ac.cn

Supplementary Figures

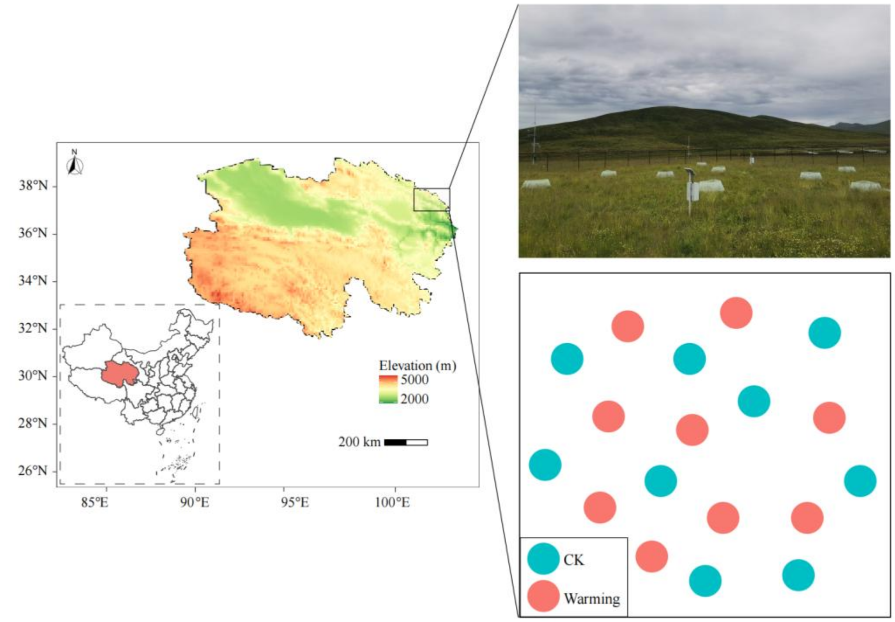

Supplementary Figure 1. Sampling information.

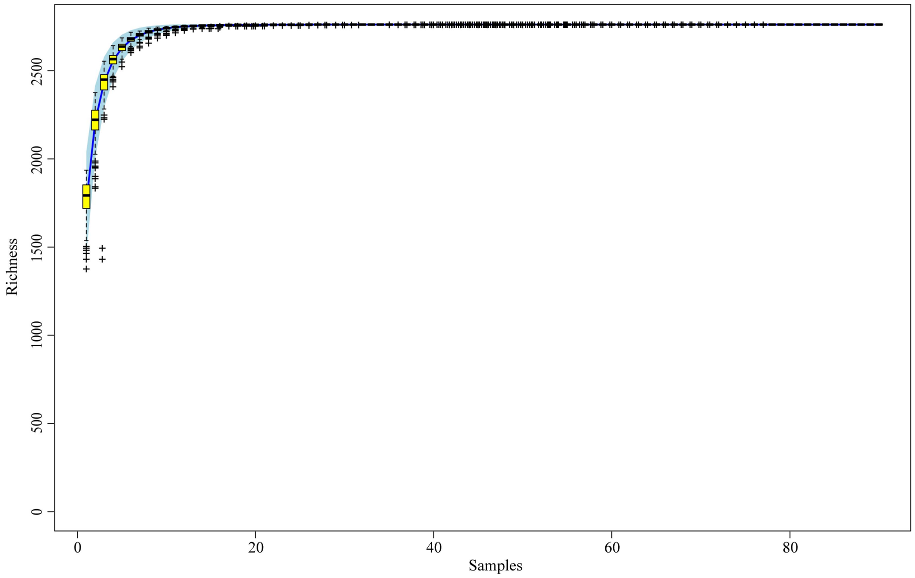

Supplementary Figure 2. Species accumulation curve.

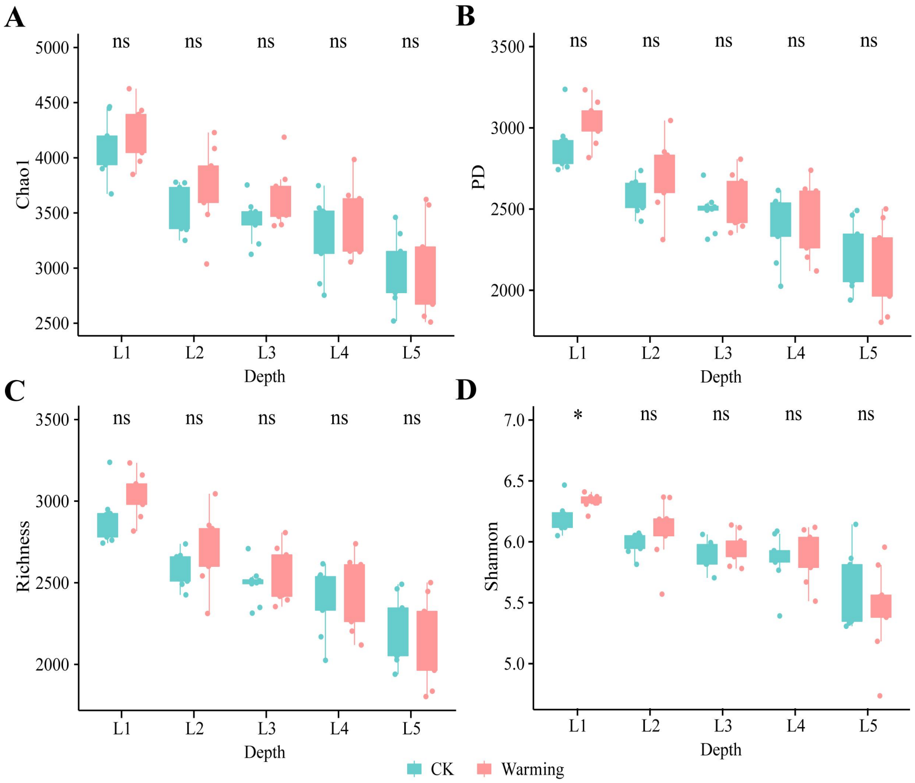

Supplementary Figure 3. The α-diversity of bacteria between control and warming at different depths (using Mann-Whitney U test)

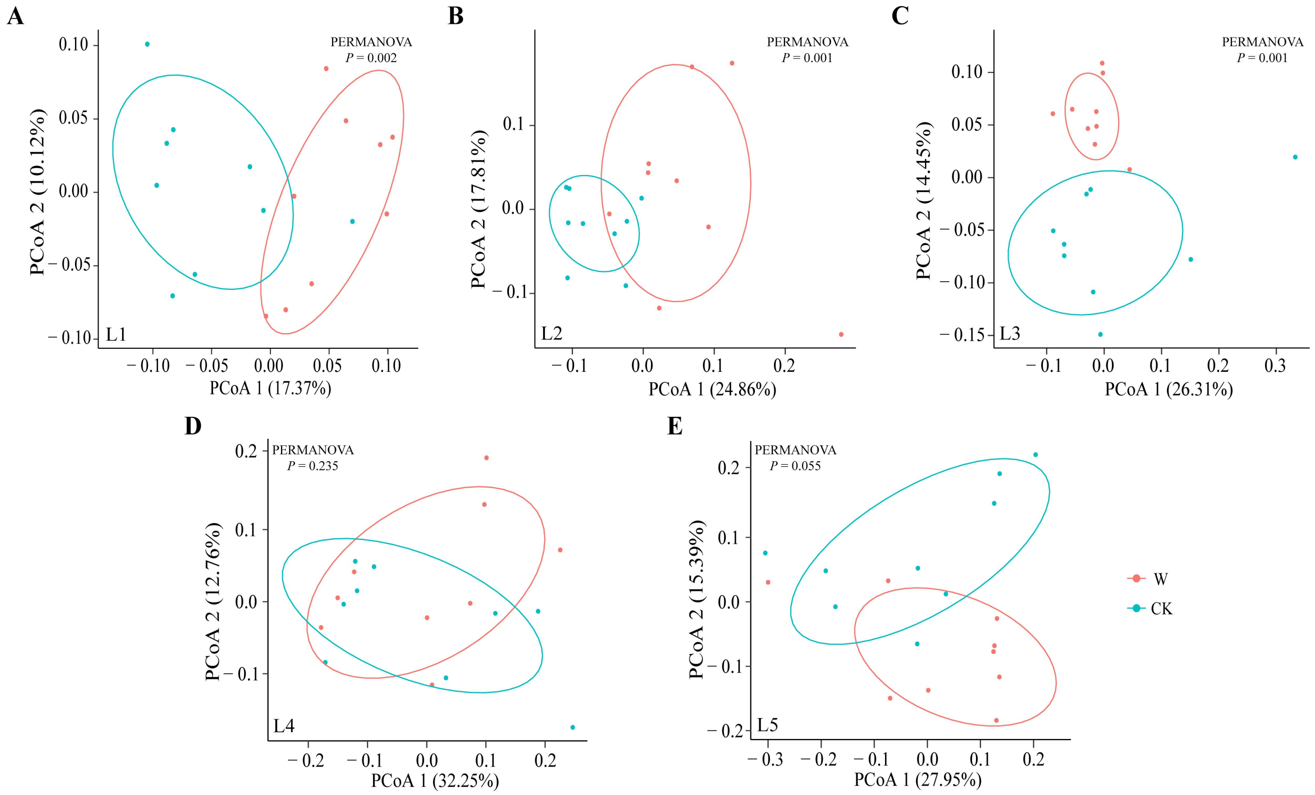

Supplementary Figure 4. PCoA analysis was performed on differences in community composition in each layer under control and warming.

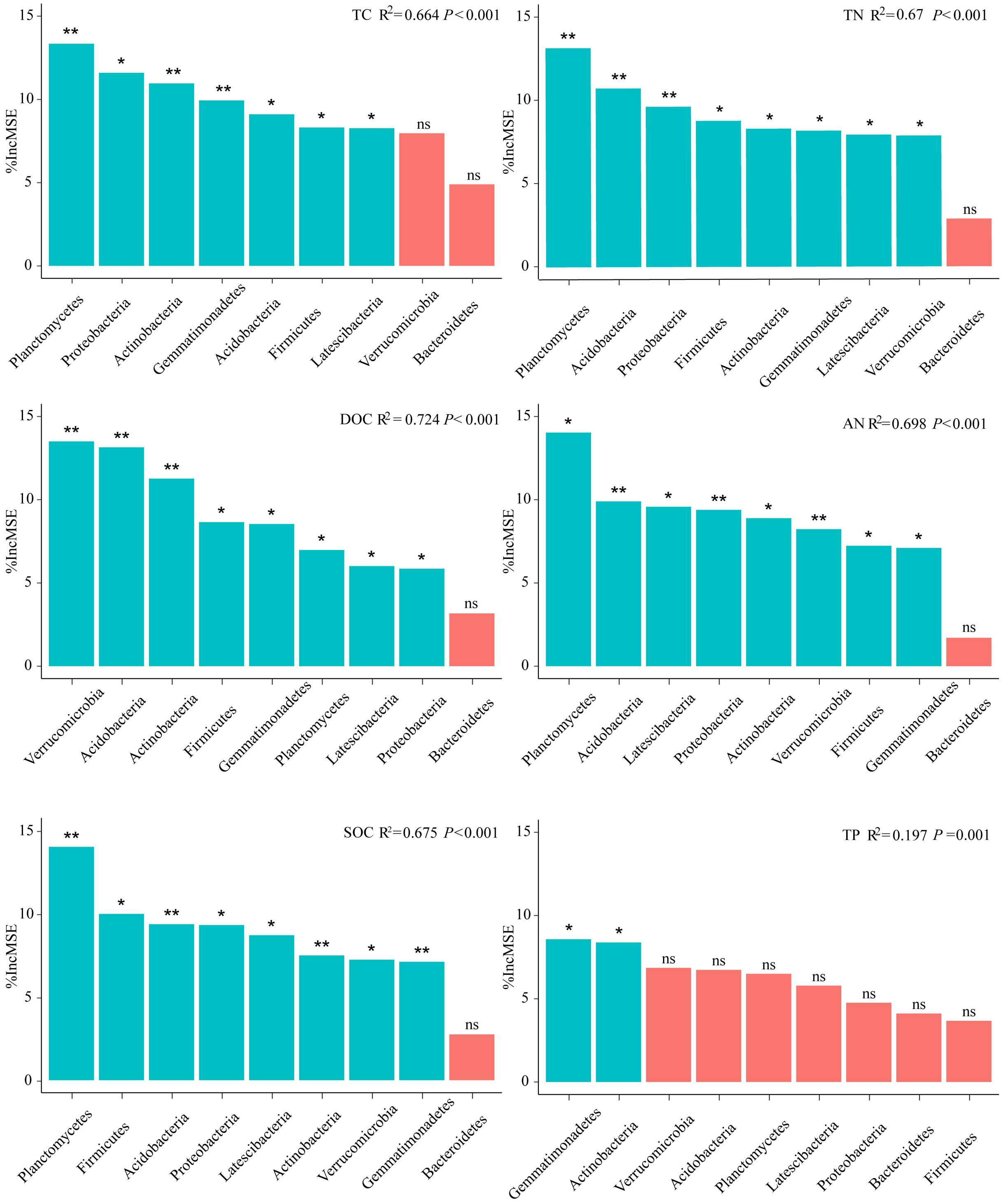

Supplementary Figure 5. Main potential bacteria contribute to soil multi-nutrient cycling in the alpine ecosystems under control. The importance of these predictors was estimated using the percent increase in the MSE of the variables, with higher MSE% values indicating more important predictors. The P < 0.05 (*) and P < 0.01 (**) indicate significance at the levels of 5% and 1%, respectively.

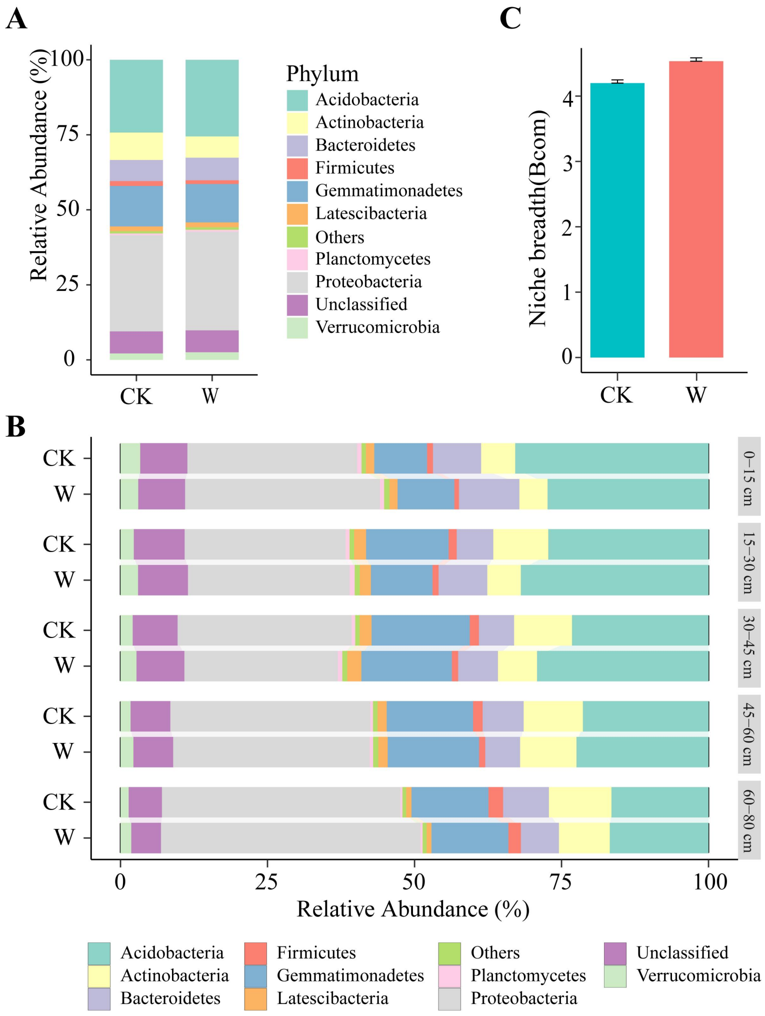

Supplementary Figure 6. The relative abundance of bacteria at the level of phylum and the bacterial niche breadth. Bacterial community composition in the whole soil profile under control and warming (A). Bacterial community composition in each layer (B). The niche breadth of soil bacteria under control and warming (C).
